# Growth Hormone as a Potential Mediator of Aerobic Exercise-Induced Reductions in Visceral Adipose Tissue

**DOI:** 10.3389/fphys.2021.623570

**Published:** 2021-04-26

**Authors:** Angelo Sabag, Dennis Chang, Nathan A. Johnson

**Affiliations:** ^1^NICM Health Research Institute, Western Sydney University, Westmead, NSW, Australia; ^2^School of Health Sciences, Faculty of Medicine and Health, The University of Sydney, Camperdown, NSW, Australia

**Keywords:** growth hormone, cardiometabolic health, abdominal adipose tissue, cardiorespiratory fitness, obesity, aging

## Introduction

Obesity remains one of the leading causes of death worldwide and is a well-known risk factor for a myriad of non-communicable diseases including diabetes, cardiovascular disease, and a variety of cancers (Wolf and Colditz, 1998; Frühbeck et al., 2013). While the relationship between obesity and cardiometabolic risk is well-established, the location of adipose tissue, particularly in the abdominal region, is considered a greater predictor of metabolic dysfunction than total fat mass (Kahn et al., 2006). Central obesity, characterized by the excess accumulation of adipose tissue in the abdominal region, is strongly and independently correlated with metabolic syndrome and is assessed clinically through the measurement of waist circumference (Shen et al., 2006). Central adiposity can be further subcategorized into abdominal subcutaneous adipose tissue (SAT) and visceral adipose tissue (VAT) (Snel et al., 2012). While the relationship between SAT and cardiometabolic risk remains equivocal, VAT has been established as a unique pathogenic fat depot. VAT acts as an endocrine organ by secreting adipocytokines and other vasoactive substances (Kanaya et al., 2004) and is associated with cardiometabolic risk independent of body mass index (BMI) or total body adiposity (Fox et al., 2007; Pak et al., 2016). Consequently, it is important to identify new, as well as further develop existing therapies to improve the management of obesity.

A landmark study in 1990 showed that exogenous growth hormone (GH) administered to older healthy males led to significant improvements in total body adiposity and lean body mass (Rudman et al., 1990). Since then, the results from further studies have shown that GH therapy can improve VAT, circulating lipid levels, and insulin resistance in adults with obesity and/or diabetes (Johannsson et al., 1997; Nam et al., 2001). Although studies like these highlighted the potential utility of GH therapy for the amelioration of age-related declines in metabolic function and body composition, further studies identified various side effects of GH therapy such as an increased likelihood of soft tissue edema, joint pain, carpal tunnel syndrome, gynecomastia, and diabetes (Liu et al., 2007). Consequently, exogenous GH therapy became typically reserved for individuals with GH deficiencies resulting from hypothalamic/pituitary disease (Clemmons et al., 2014). Despite this, there has since been increasing interest in identifying therapies, including lifestyle interventions, that increase physiologic GH release and action.

Exercise and diet modification are cornerstone therapies for the management of obesity-related disease. Interestingly, pooled data from clinical trials show that while exercise is less effective than diet modification for body weight loss, it appears to elicit superior reductions in VAT (Verheggen et al., 2016). This finding may partly be explained by exercise-induced changes in lipolytic hormones, such as GH, during and after exercise, which seem to target VAT (Berryman and List, 2017). Acute exercise has been shown to temporarily increase GH release in an intensity-dependent manner (Godfrey et al., 2003), and such responses appear to be mediated by cardiorespiratory fitness (CRF) (Holt et al., 2001). However, the degree to which temporal exercise-induced changes in GH release and action improve VAT is unclear and warrants further investigation. Furthermore, although both aerobic and resistance exercise elicit a GH response, the relative contribution of aerobic exercise on GH response and action arguably is less clear. Consequently, this article will evaluate the various factors that contribute to aerobic exercise-induced GH response and how these changes influence VAT and cardiometabolic health more broadly.

## Somatotropic Axis

The somatotropic axis is a primary regulator of metabolism and consists of GH and insulin-like growth factors (IGF-I and -II), and their associated carrier proteins and receptors, which are further regulated by nutritional status and hormones such as ghrelin and insulin (Renaville et al., 2002; Savastano et al., 2014). GH is secreted at the anterior pituitary gland in a pulsatile manner and is primarily regulated by hypothalamic neuropeptides GH-releasing hormone (GHRH) and somatostatin, which stimulate and inhibit GH secretion, respectively (Vijayakumar et al., 2011). GH affects multiple systems within the body and is the primary secretagogue of IGF-I, which itself is a regulator of GH secretion (Ohlsson et al., 2009).

GH is a potent anabolic hormone that plays a significant role in lipid metabolism at various sites including the liver, skeletal muscle, and adipose tissue (Dehkhoda et al., 2018). During periods of fasting or stress, GH promotes the use of lipids as the primary fuel source in order to preserve carbohydrates and protein stores (Lewitt, 2017). In the liver, lipid uptake and production are increased through the phosphorylation of sterol regulatory element-binding proteins (SREBP-1a) and by increased lipoprotein lipase (LPL) expression (Vijayakumar et al., 2011). In addition to this, GH also indirectly increases fatty acid oxidation and activates the adenosine monophosphate-activated protein kinase (AMPK) pathway (Vijayakumar et al., 2011).

While GH is a powerful regulator of lipid metabolism, its role varies depending on the target site. For example, although GH has lipogenic effects within the liver, the opposite occurs in adipose tissue, particularly VAT, where GH elicits lipolytic effects through the suppression of LPL activity (Stanley and Grinspoon, 2015). During exercise or fasting, GH stimulates the release of free fatty acids (FFAs) into circulation where they are transported to various organs, including myocytes where they may be repackaged as triglycerides or undergo β-oxidation in the mitochondria. While it is recognized that GH also elicits various effects on glucose and protein metabolism, exercise-induced alterations in physiologic GH appear to primarily affect lipid metabolism (Kanaley et al., 2004).

## Obesity

The bidirectional relationship between central obesity and impaired GH secretion has been widely reported despite being scantily understood (Stanley and Grinspoon, 2015; Lewitt, 2017). Increased ectopic fat, such as VAT and intrahepatic triglyceride, contributes to insulin resistance and may affect the feedback control system of the somatotropic axis, resulting in a cascade of metabolic impairments (Savastano et al., 2014). Interestingly, physiologic increases in GH secretion through fasting or exercise contribute to increases in circulating FFAs; however, these do not lead to metabolic impairments due to various mechanisms, such as concurrent increases in skeletal muscle fatty acid uptake and oxidation (Huang et al., 2020). Interestingly, a study by Stokes et al. (2008) showed that FFA levels may also regulate GH *via* a negative feedback control, as nicotinic acid-mediated suppression of lipolysis, and consequently reduced circulating FFAs, led to a significantly greater GH response. This finding may help further explain why individuals with obesity and reduced CRF, who on average have elevated levels of FFAs (König et al., 2003; Boden, 2008), exhibit impaired GH secretion and action, as their ability to uptake and oxidize FFAs is reduced (Kim et al., 2000).

The obesity phenotype shares considerable overlapping risk factors with adult GH deficiency such as increased serum low-density lipoprotein cholesterol (Cordido et al., 2010) and inflammation (Utz et al., 2008), thereby making it difficult to decouple the cause from the effect. However, it is likely that impaired GH secretion is an acquired transient defect that occurs prior to the onset of obesity, as previous reports showed that following 2 weeks of overeating, GH levels were decreased while body weight remained unchanged (Cornford et al., 2011). Importantly, not all adult GH deficiency is caused by obesity-inducing behaviors, as adults with hypothalamic or pituitary diseases also exhibit suppressed GH production and increased central adiposity. A known therapy for improving many of the aforementioned risk factors is energy restriction *via* diet modification. In fact, a previous study involving 18 adults with obesity and 18 age- and sex-matched controls showed that defects in GH secretion were ameliorated to near-normal function following significant diet-induced weight loss (Rasmussen et al., 1995). Other lifestyle interventions such as exercise have been shown to alter physiologic GH response; however, as mentioned previously, this response has been shown to be blunted in people with obesity, including childhood obesity (Oliver et al., 2012). These data suggest that obesity-inducing behaviors and obesity itself both contribute to altered GH release and function beyond normal age-related declines.

## Aerobic Exercise

Regular aerobic exercise enhances the body's ability to transport and oxidize FFAs during exercise at varying work rates (Van Tienen et al., 2012). This is also true for individuals with impaired fatty acid oxidation, such as those with obesity (Kim et al., 2000) and diabetes (Ghanassia et al., 2006; Van Tienen et al., 2012), and it has been suggested that these improvements may be mediated through exercise-induced increases in mitochondrial and fatty acid transporter content, carnitine shuttle activity (Melanson et al., 2009), and cardiorespiratory fitness (Kujala et al., 2019). In fact, low CRF may be a greater predictor of metabolic dysfunction than VAT (Kim et al., 2014), and as such, improving CRF has emerged as a therapeutic target for individuals with obesity-related disease, which may also serve to reverse GH-related impairments.

As mentioned earlier, GH promotes lipolysis within adipose tissue and increases mitochondrial oxidative capacity (Short et al., 2008). Although obesity blunts exercise-induced GH response, CRF, which in part reflects muscle oxidative capacity, appears to be a greater determinant of exercise-induced GH secretion than obesity (Holt et al., 2001). Therefore, at least in theory, improving CRF in the absence of weight loss should still yield beneficial effects of GH secretion. However, despite exercise and GH eliciting similar effects on adipose tissue and lipid metabolism, it is unclear whether exercise-induced improvements in central adiposity are mediated by concurrent changes in physiologic GH response or if the improvements in central adiposity and GH response are simply independent by-products of exercise adherence. As GH responses to acute and chronic exercise are affected by a variety of factors such CRF, exercise volume, and exercise intensity (Frystyk, 2010), further research is required to determine optimal exercise prescriptions for the amelioration of somatotropic dysfunction.

Acute aerobic exercise has been shown to increase GH levels, and these changes have been shown to be strongly associated with exercise intensity and volume, a function of exercise duration and frequency (Pritzlaff et al., 1999). In fact, current available evidence suggests that exercise-induced GH responses may only be elicited at or above specific exercise volume and intensity parameters. For example, Felsing et al. (1992) showed that in order to increase circulating GH, healthy adult men needed to exercise for a minimum of 10 min above lactate threshold. Similarly, Gilbert et al. (2008) showed that healthy adult men performing 30 min of aerobic exercise at 70% VO_2peak_ elicited greater GH response than when performing a single bout of 30-s sprint on a cycle ergometer. While the majority of research has been undertaken in men, a study by Sauro and Kanaley (2003) also showed that exercising at 75% VO_2peak_ for a minimum of 10 min is sufficient to increase GH response in healthy young women. These findings highlight that while exercise intensity does appear to influence GH levels acutely, sufficient exercise volume may also be required.

Currently, it is unclear whether regular aerobic exercise can increase the GH-response to acute exercise. A study by Sasaki et al. (2014) reported that following 4 weeks of high-intensity interval training (HIIT) or moderate-intensity continuous training (MICT), the magnitude of GH response to exercise did not increase from pre-intervention measures in sedentary but otherwise healthy men for both interventions. Furthermore, neither HIIT nor MICT improved whole-body fat mass, liver fat, and intramyocellular lipid content. Further findings from a recent randomized controlled trial involving young women with obesity showed that when compared to energy-matched MICT, HIIT or supramaximal aerobic exercise led to greater VAT reduction but not greater changes from pre-intervention levels in serum GH measured immediately or 4 h after exercise (Zhang et al., 2021). In fact, all groups showed elevated GH responses to exercise; however, only the higher-intensity interventions decreased VAT, suggesting that other factors likely contributed to these improvements in this cohort.

While there are limited data pertaining to the effects of regular exercise on chronic changes in 24-h GH release and function, a study involving untrained eumenorrheic women showed that regular aerobic exercise above lactate threshold led to a two-fold increase in 24-h GH secretion (Weltman et al., 1992). A recent study involving young and middle-aged men showed that regular concurrent resistance and aerobic exercise led to significantly increased GH levels at rest (Sellami et al., 2017); however, whether these changes influence 24-h GH release remains unknown. Another limitation of the GH and exercise literature is that most studies are undertaken in younger to middle-aged adults. Consequently, it is unclear whether these findings translate to older adults or those with more severe metabolic abnormalities. A recent review involving three studies showed that regular exercise resulted in negligible effects on GH in older adults (Sellami et al., 2019). As the studies included in the review were limited to small samples and did not include stand-alone aerobic exercise interventions, further research is required to determine whether exercise-induced changes in physiologic GH improve age- and obesity-related disease in elderly cohorts. Furthermore, there is limited data pertaining to the effects of chronic exercise on basal GH or 24-h GH release; therefore, we are unable to comment on whether any such changes would increase total energy expenditure or fat oxidation rates.

**Figure 1:**
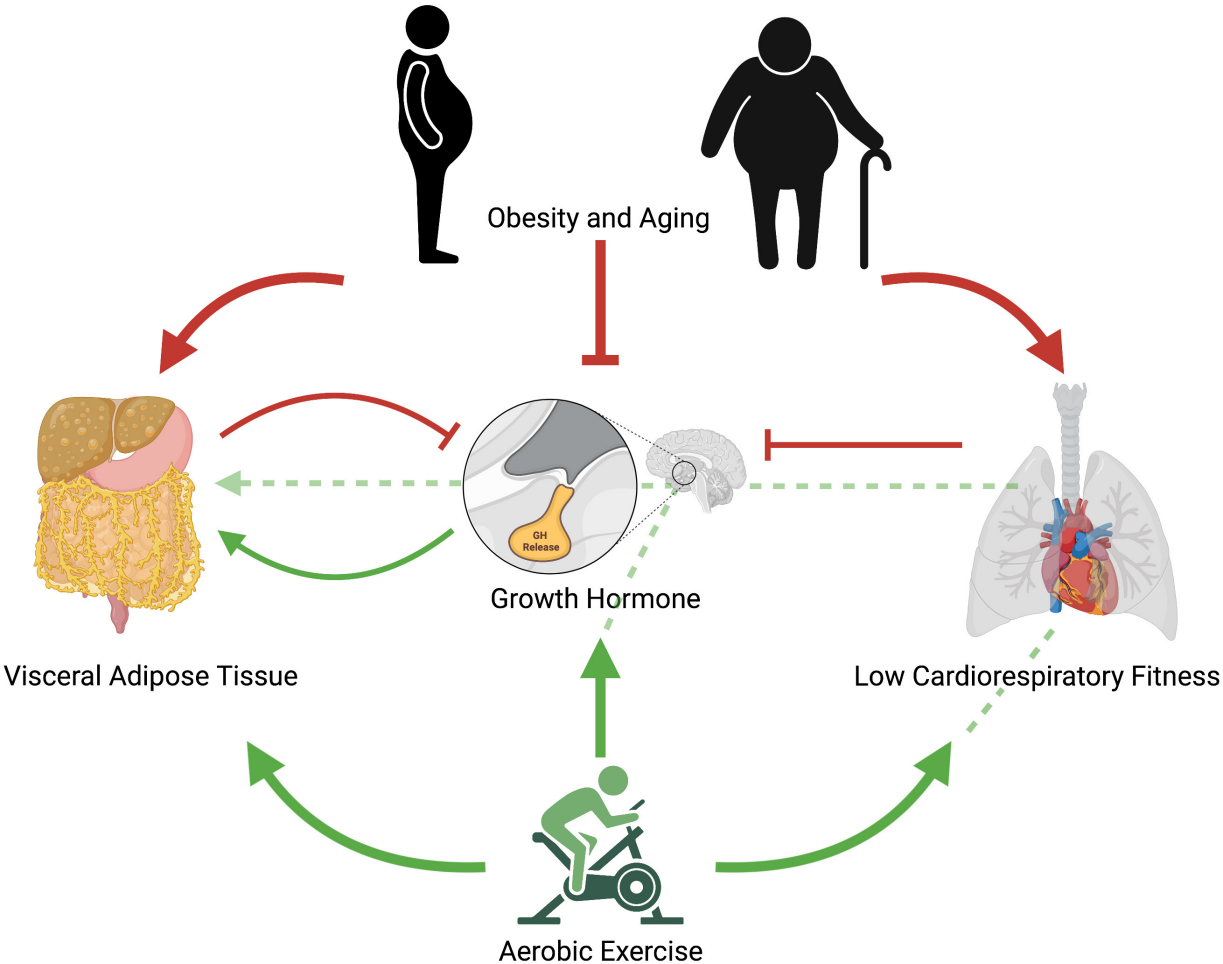
Proposed mechanistic pathway for aerobic exercise induced changes in growth hormone release and ensuing reductions in visceral adipose tissue. Red arrows represent negative effects; green arrows represent positive effects; unbroken red lines represent inhibitory effects; broken green lines represent potential positive pathways of exercise-induced improvements in cardiometabolic health. Obesity and aging contribute to increased visceral adipose tissue, reduced cardiorespiratory fitness, and impaired growth hormone release. Low cardiorespiratory fitness further contributes to impaired growth hormone release as does visceral adipose tissue. Regular aerobic exercise reduces visceral adipose tissue, increases growth hormone release, and increases cardiorespiratory fitness. Regular aerobic exercise may also indirectly improve visceral adipose tissue through increases in cardiorespiratory fitness, which lead to improved growth hormone response and ensuing interactions in visceral adipocytes. Acute aerobic exercise elicits growth hormone release, which increases lipolysis of visceral adipose tissue.

Although the precise mechanisms driving exercise-induced improvements in cardiometabolic outcomes remain a matter of scientific debate, given the congruent metabolic effects of exercise, particularly aerobic exercise, and GH, it could follow that the behavior itself and ensuing endocrine and metabolic changes both improve cardiometabolic health, with the latter providing somewhat of an additive, but perhaps not essential, effect (Figure 1). Like GH, exercise exerts potent lipolytic effects, particularly on VAT (Tsiloulis and Watt, 2015). Furthermore, exercise has been shown to elicit significant improvements in ectopic fat in the absence of weight loss (Sabag et al., 2017, 2020), thus indicating that these improvements are mediated through mechanisms other than simple energy expenditure. As exercise-induced GH response occurs in an intensity-dependent manner, and appears to be mediated by CRF, this may explain why HIIT can lead to similar improvements in waist circumference and VAT than in higher-volume MICT despite requiring less time and expending less energy (Keating et al., 2015). While the current evidence remains circumstantial, future research exploring the relative contribution of somatotropic change in exercise-related metabolic improvements could have far-reaching implications, including advancing current exercise prescription practices for the management of cardiometabolic health.

## Conclusion

Based on the current available literature, it appears that exercise-induced reductions in VAT are mediated by multiple factors, which may include acute and chronic exercise-induced change in GH levels. This could be due to the similar lipolytic effects of both GH and exercise on VAT. Furthermore, because CRF plays a significant role in GH response, partaking in regular exercise may ameliorate age-related reductions in GH response and action.

As exercise has been shown to ameliorate obesity-related CRF and other cardiometabolic impairments, exercise should be incorporated into routine care for the treatment of adult-onset GH deficiency and associated metabolic perturbations. Importantly, increasing CRF and weight loss concurrently through exercise and dietary modification may yield greater restoration of GH function than either intervention on its own; however, there is limited evidence to confirm this. Consequently, further research is required to elucidate the relationship between somatotropic changes and exercise-induced cardiometabolic improvements.

## Author Contributions

The conception and drafting of this article was led by AS. NJ and DC contributed to the drafting and critical revision of the article. All authors approved the submitted version.

## Conflict of Interest

The authors declare that the research was conducted in the absence of any commercial or financial relationships that could be construed as a potential conflict of interest.

## References

[B1] Berryman D. E. List E. O. (2017). Growth hormone's effect on adipose tissue: quality versus quantity. Int. J. Mol. Sci. 18:1621. 10.3390/ijms18081621 28933734 PMC5578013

[B2] Boden G. (2008). Obesity and free fatty acids. Endocrinol. Metab. Clin. North Am. 37, 635–646. 10.1016/j.ecl.2008.06.007 18775356 PMC2596919

[B3] Clemmons D. R. Molitch M. Hoffman A. R. Klibanski A. Strasburger C. J. Kleinberg D. L.. (2014). Growth hormone should be used only for approved indications. J. Clin. Endocrinol. Metab. 99, 409–411. 10.1210/jc.2013-4187 24423315 PMC5393472

[B4] Cordido F. Garcia-Buela J. Sangiao-Alvarellos S. Martinez T. Vidal O. (2010). The decreased growth hormone response to growth hormone releasing hormone in obesity is associated to cardiometabolic risk factors. Mediators Inflamm. 2010:434562. 10.1155/2010/434562 20150954 PMC2817384

[B5] Cornford A. S. Barkan A. L. Horowitz J. F. (2011). Rapid suppression of growth hormone concentration by overeating: potential mediation by hyperinsulinemia. J. Clin. Endocrinol. Metab. 96, 824–830. 10.1210/jc.2010-1895 21209037 PMC3047219

[B6] Dehkhoda F. Lee C. M. M. Medina J. Brooks A. J. (2018). The growth hormone receptor: mechanism of receptor activation, cell signaling, and physiological aspects. Front. Endocrinol. (Lausanne) 9:35. 10.3389/fendo.2018.00035 29487568 PMC5816795

[B7] Felsing N. E. Brasel J. A. Cooper D. M. (1992). Effect of low and high intensity exercise on circulating growth hormone in men. J. Clin. Endocrinol. Metab. 75, 157–162. 10.1210/jc.75.1.157 1619005

[B8] Fox C. S. Massaro J. M. Hoffmann U. Pou K. M. Maurovich-Horvat P. Liu C. Y.. (2007). Abdominal visceral and subcutaneous adipose tissue compartments: association with metabolic risk factors in the Framingham Heart Study. Circulation 116, 39–48. 10.1161/CIRCULATIONAHA.106.675355 17576866

[B9] Frühbeck G. Toplak H. Woodward E. Yumuk V. Maislos M. Oppert J. M. (2013). Obesity: the gateway to ill health–an EASO position statement on a rising public health, clinical, and scientific challenge in Europe. Obes. Facts 6, 117–120. 10.1159/000350627 23548858 PMC5644725

[B10] Frystyk J. (2010). Exercise and the growth hormone-insulin-like growth factor axis. Med. Sci. Sports Exerc. 42, 58–66. 10.1249/MSS.0b013e3181b07d2d 20010129

[B11] Ghanassia E. Brun J. F. Fedou C. Raynaud E. Mercier J. (2006). Substrate oxidation during exercise: type 2 diabetes is associated with a decrease in lipid oxidation and an earlier shift towards carbohydrate utilization. Diabetes Metab. 32, 604–610. 10.1016/S1262-3636(07)70315-4 17296514

[B12] Gilbert K. L. Stokes K. A. Hall G. M. Thompson D. (2008). Growth hormone responses to 3 different exercise bouts in 18- to 25- and 40- to 50-year-old men. Appl. Physiol. Nutr. Metab. 33, 706–712. 10.1139/H08-034 18641713

[B13] Godfrey R. J. Madgwick Z. Whyte G. P. (2003). The exercise-induced growth hormone response in athletes. Sports Med. 33, 599–613. 10.2165/00007256-200333080-00005 12797841

[B14] Holt R. I. Webb E. Pentecost C. Sönksen P. H. (2001). Aging and physical fitness are more important than obesity in determining exercise-induced generation of GH. J. Clin. Endocrinol. Metab. 86, 5715–5720. 10.1210/jcem.86.12.8092 11739427

[B15] Huang Z. Huang L. Waters M. J. Chen C. (2020). Insulin and growth hormone balance: implications for obesity. Trends Endocrinol. Metab. 31, 642–654. 10.1016/j.tem.2020.04.005 32416957

[B16] Johannsson G. Mårin P. Lönn L. Ottosson M. Stenlöf K. Björntorp P.. (1997). Growth hormone treatment of abdominally obese men reduces abdominal fat mass, improves glucose and lipoprotein metabolism, and reduces diastolic blood pressure. J. Clin. Endocrinol. Metab. 82, 727–734. 10.1210/jcem.82.3.3809 9062473

[B17] Kahn S. E. Hull R. L. Utzschneider K. M. (2006). Mechanisms linking obesity to insulin resistance and type 2 diabetes. Nature 444, 840–846. 10.1038/nature05482 17167471

[B18] Kanaley J. A. Dall R. Møller N. Nielsen S. C. Christiansen J. S. Jensen M. D.. (2004). Acute exposure to GH during exercise stimulates the turnover of free fatty acids in GH-deficient men. J. Appl. Physiol. (1985) 96, 747–753. 10.1152/japplphysiol.00711.2003 14594860

[B19] Kanaya A. M. Harris T. Goodpaster B. H. Tylavsky F. Cummings S. R. (2004). Adipocytokines attenuate the association between visceral adiposity and diabetes in older adults. Diabetes Care 27, 1375–1380. 10.2337/diacare.27.6.1375 15161791

[B20] Keating S. E. Hackett D. A. Parker H. M. O'connor H. T. Gerofi J. A. Sainsbury A.. (2015). Effect of aerobic exercise training dose on liver fat and visceral adiposity. J. Hepatol. 63, 174–182. 10.1016/j.jhep.2015.02.022 25863524

[B21] Kim J. Y. Hickner R. C. Cortright R. L. Dohm G. L. Houmard J. A. (2000). Lipid oxidation is reduced in obese human skeletal muscle. Am. J. Physiol. Endocrinol. Metab. 279, E1039–E1044. 10.1152/ajpendo.2000.279.5.E1039 11052958

[B22] Kim S. Kim J. Y. Lee D. C. Lee H. S. Lee J. W. Jeon J. Y. (2014). Combined impact of cardiorespiratory fitness and visceral adiposity on metabolic syndrome in overweight and obese adults in Korea. PLoS ONE 9:e85742. 10.1371/journal.pone.0085742 24454926 PMC3893257

[B23] König D. Väisänen S. B. Bouchard C. Halle M. Lakka T. A. Baumstark M. W.. (2003). Cardiorespiratory fitness modifies the association between dietary fat intake and plasma fatty acids. Eur. J. Clin. Nutr. 57, 810–815. 10.1038/sj.ejcn.1601613 12821879

[B24] Kujala U. M. Vaara J. P. Kainulainen H. Vasankari T. Vaara E. Kyröläinen H. (2019). Associations of aerobic fitness and maximal muscular strength with metabolites in young men. JAMA Netw. Open 2:e198265. 10.1001/jamanetworkopen.2019.8265 31441934 PMC6714035

[B25] Lewitt M. S. (2017). The role of the growth hormone/insulin-like growth factor system in visceral adiposity. Biochem. Insights 10:1178626417703995–1178626417703995. 10.1177/1178626417703995 28469442 PMC5404904

[B26] Liu H. Bravata D. M. Olkin I. Nayak S. Roberts B. Garber A. M.. (2007). Systematic review: the safety and efficacy of growth hormone in the healthy elderly. Ann. Intern. Med. 146, 104–115. 10.7326/0003-4819-146-2-200701160-00005 17227934

[B27] Melanson E. L. Maclean P. S. Hill J. O. (2009). Exercise improves fat metabolism in muscle but does not increase 24-h fat oxidation. Exerc. Sport Sci. Rev. 37, 93–101. 10.1097/JES.0b013e31819c2f0b 19305201 PMC2885974

[B28] Nam S. Y. Kim K. R. Cha B. S. Song Y. D. Lim S. K. Lee H. C.. (2001). Low-dose growth hormone treatment combined with diet restriction decreases insulin resistance by reducing visceral fat and increasing muscle mass in obese type 2 diabetic patients. Int. J. Obes. Relat. Metab. Disord. 25, 1101–1107. 10.1038/sj.ijo.0801636 11477493

[B29] Ohlsson C. Mohan S. SjöGren K. Tivesten A. S. Isgaard J. R. Isaksson O.. (2009). The role of liver-derived insulin-like growth factor-I. Endocr. Rev. 30, 494–535. 10.1210/er.2009-0010 19589948 PMC2759708

[B30] Oliver S. R. Hingorani S. R. Rosa J. S. Zaldivar F. P. Galassetti P. R. (2012). Synergistic effect of obesity and lipid ingestion in suppressing the growth hormone response to exercise in children. J. Appl. Physiol. (1985) 113, 192–198. 10.1152/japplphysiol.01184.2011 22518832 PMC3404705

[B31] Pak K. Lee S. H. Lee J. G. Seok J. W. Kim I. J. (2016). Comparison of visceral fat measures with cardiometabolic risk factors in healthy adults. PLoS ONE 11:e0153031. 10.1371/journal.pone.0153031 27043708 PMC4820273

[B32] Pritzlaff C. J. Wideman L. Weltman J. Y. Abbott R. D. Gutgesell M. E. Hartman M. L.. (1999). Impact of acute exercise intensity on pulsatile growth hormone release in men. J. Appl. Physiol. (1985) 87, 498–504. 10.1152/jappl.1999.87.2.498 10444604

[B33] Rasmussen M. H. Hvidberg A. Juul A. Main K. M. Gotfredsen A. Skakkebaek N. E.. (1995). Massive weight loss restores 24-hour growth hormone release profiles and serum insulin-like growth factor-I levels in obese subjects. J. Clin. Endocrinol. Metab. 80, 1407–1415. 10.1210/jcem.80.4.7536210 7536210

[B34] Renaville R. Hammadi M. Portetelle D. (2002). Role of the somatotropic axis in the mammalian metabolism. Domest. Anim. Endocrinol. 23, 351–360. 10.1016/S0739-7240(02)00170-4 12142251

[B35] Rudman D. Feller A. G. Nagraj H. S. Gergans G. A. Lalitha P. Y. Goldberg A. F.. (1990). Effects of human growth hormone in men over 60 years old. N. Engl. J. Med. 323, 1–6. 10.1056/NEJM199007053230101 2355952

[B36] Sabag A. Way K. L. Keating S. E. Sultana R. N. O'connor H. T. Baker M. K.. (2017). Exercise and ectopic fat in type 2 diabetes: a systematic review and meta-analysis. Diabetes Metab. 43, 195–210. 10.1016/j.diabet.2016.12.006 28162956

[B37] Sabag A. Way K. L. Sultana R. N. Keating S. E. Gerofi J. A. Chuter V. H.. (2020). The effect of a novel low-Volume aerobic exercise intervention on liver fat in type 2 diabetes: a randomized controlled trial. Diabetes Care 43, 2371–2378. 10.2337/dc19-2523 32732374

[B38] Sasaki H. Morishima T. Hasegawa Y. Mori A. Ijichi T. Kurihara T.. (2014). 4 weeks of high-intensity interval training does not alter the exercise-induced growth hormone response in sedentary men. Springerplus 3:336. 10.1186/2193-1801-3-336 25806146 PMC4363223

[B39] Sauro L. M. Kanaley J. A. (2003). The effect of exercise duration and mode on the growth hormone responses in young women on oral contraceptives. Eur. J. Appl. Physiol. 90, 69–75. 10.1007/s00421-003-0863-x 12811570

[B40] Savastano S. Di Somma C. Barrea L. Colao A. (2014). The complex relationship between obesity and the somatropic axis: the long and winding road. Growth Horm. IGF Res. 24, 221–226. 10.1016/j.ghir.2014.09.002 25315226

[B41] Sellami M. Bragazzi N. L. Slimani M. Hayes L. Jabbour G. De Giorgio A.. (2019). The effect of exercise on glucoregulatory hormones: a countermeasure to human aging: insights from a comprehensive review of the literature. Int. J. Environ. Res. Public Health 16:1709. 10.3390/ijerph16101709 31096708 PMC6572009

[B42] Sellami M. Dhahbi W. Hayes L. D. Padulo J. Rhibi F. Djemail H.. (2017). Combined sprint and resistance training abrogates age differences in somatotropic hormones. PLoS ONE 12:e0183184. 10.1371/journal.pone.0183184 28800636 PMC5553853

[B43] Shen W. Punyanitya M. Chen J. Gallagher D. Albu J. Pi-Sunyer X.. (2006). Waist circumference correlates with metabolic syndrome indicators better than percentage fat. Obesity (Silver Spring) 14, 727–736. 10.1038/oby.2006.83 16741276 PMC1894647

[B44] Short K. R. Moller N. Bigelow M. L. Coenen-Schimke J. Nair K. S. (2008). Enhancement of muscle mitochondrial function by growth hormone. J. Clin. Endocrinol. Metab. 93, 597–604. 10.1210/jc.2007-1814 18000087 PMC2243230

[B45] Snel M. Jonker J. T. Schoones J. Lamb H. De Roos A. Pijl H.. (2012). Ectopic fat and insulin resistance: pathophysiology and effect of diet and lifestyle interventions. Int. J. Endocrinol. 2012:983814. 10.1155/2012/983814 22675355 PMC3366269

[B46] Stanley T. L. Grinspoon S. K. (2015). Effects of growth hormone-releasing hormone on visceral fat, metabolic, and cardiovascular indices in human studies. Growth Horm. IGF Res. 25, 59–65. 10.1016/j.ghir.2014.12.005 25555516 PMC4324360

[B47] Stokes K. A. Tyler C. Gilbert K. L. (2008). The growth hormone response to repeated bouts of sprint exercise with and without suppression of lipolysis in men. J. Appl. Physiol. (1985) 104, 724–728. 10.1152/japplphysiol.00534.2007 18187617

[B48] Tsiloulis T. Watt M. J. (2015). Exercise and the regulation of adipose tissue metabolism. Prog. Mol. Biol. Transl. Sci. 135, 175–201. 10.1016/bs.pmbts.2015.06.016 26477915

[B49] Utz A. L. Yamamoto A. Hemphill L. Miller K. K. (2008). Growth hormone deficiency by growth hormone releasing hormone-arginine testing criteria predicts increased cardiovascular risk markers in normal young overweight and obese women. J. Clin. Endocrinol. Metab. 93, 2507–2514. 10.1210/jc.2008-0169 18445664 PMC2453050

[B50] Van Tienen F. H. Praet S. F. De Feyter H. M. Van Den Broek N. M. Lindsey P. J. Schoonderwoerd K. G.. (2012). Physical activity is the key determinant of skeletal muscle mitochondrial function in type 2 diabetes. J. Clin. Endocrinol. Metab. 97, 3261–3269. 10.1210/jc.2011-3454 22802091

[B51] Verheggen R. J. Maessen M. F. Green D. J. Hermus A. R. Hopman M. T. Thijssen D. H. (2016). A systematic review and meta-analysis on the effects of exercise training versus hypocaloric diet: distinct effects on body weight and visceral adipose tissue. Obes. Rev. 17, 664–690. 10.1111/obr.12406 27213481

[B52] Vijayakumar A. Yakar S. Leroith D. (2011). The intricate role of growth hormone in metabolism. Front. Endocrinol. (Lausanne) 2:32. 10.3389/fendo.2011.00032 22654802 PMC3356038

[B53] Weltman A. Weltman J. Y. Schurrer R. Evans W. S. Veldhuis J. D. Rogol A. D. (1992). Endurance training amplifies the pulsatile release of growth hormone: effects of training intensity. J. Appl. Physiol. (1985) 72, 2188–2196. 10.1152/jappl.1992.72.6.2188 1629072

[B54] Wolf A. M. Colditz G. A. (1998). Current estimates of the economic cost of obesity in the United States. Obes. Res. 6, 97–106. 10.1002/j.1550-8528.1998.tb00322.x 9545015

[B55] Zhang H. Tong T. K. Kong Z. Shi Q. Liu Y. Nie J. (2021). Exercise training-induced visceral fat loss in obese women: the role of training intensity and modality. Scand. J. Med. Sci. Sports 31, 30–43. 10.1111/sms.13803 32789898

